# Effects of Physical Exercise Breaks on Executive Function in a Simulated Classroom Setting: Uncovering a Window into the Brain

**DOI:** 10.1002/advs.202406631

**Published:** 2024-11-25

**Authors:** Qian Yu, Zhihao Zhang, Sebastian Ludyga, Kirk I. Erickson, Boris Cheval, Meijun Hou, Dominika M. Pindus, Charles H. Hillman, Arthur F. Kramer, Ryan S. Falck, Teresa Liu‐Ambrose, Jin Kuang, Sean P. Mullen, Keita Kamijo, Toru Ishihara, David A. Raichlen, Matthew Heath, David Moreau, André O. Werneck, Fabian Herold, Liye Zou

**Affiliations:** ^1^ Body‐Brain‐Mind Laboratory School of Psychology Shenzhen University Shenzhen 518060 China; ^2^ Faculty of Education University of Macau Macau 999078 China; ^3^ Department of Sport, Exercise, and Health University of Basel Basel 4052 Switzerland; ^4^ AdventHealth Research Institute, Neuroscience Orlando FL 32101 USA; ^5^ Department of Psychology University of Pittsburgh Pittsburgh 15260 USA; ^6^ Department of Sport Sciences and Physical Education Ecole Normale Supérieure Rennes Bruz 35170 France; ^7^ Laboratory VIPS2 University of Rennes Rennes 35042 France; ^8^ Department of Health and Kinesiology University of Illinois at Urbana‐Champaign Urbana IL 61801 USA; ^9^ Beckman Institute for Advanced Science and Technology University of Illinois at Urbana‐Champaign Urbana IL 61801 USA; ^10^ Neuroscience Program University of Illinois at Urbana‐Champaign Urbana IL 61801 USA; ^11^ Center for Cognitive and Brain Health Northeastern University Boston MA USA; ^12^ Department of Physical Therapy, Movement, and Rehabilitation Sciences Northeastern University Boston MA 02115 USA; ^13^ Department of Psychology Northeastern University Boston MA 02115 USA; ^14^ Department of Physical Therapy Faculty of Medicine University of British Columbia Vancouver British Columbia V6T 1Z4 Canada; ^15^ Centre for Aging SMART at Vancouver Coastal Health Vancouver Coastal Health Research Institute University of British Columbia Vancouver, British Columbia V6T 1Z4 Canada; ^16^ Center for Social & Behavioral Science University of Illinois, Urbana‐Champaign Champaign 61820 USA; ^17^ Informatics Programs University of Illinois, Urbana‐Champaign Champaign 61820 USA; ^18^ Faculty of Liberal Arts and Sciences Chukyo University Nagoya 466‐8666 Japan; ^19^ Graduate School of Human Development and Environment Kobe University Kobe 657‐8501 Japan; ^20^ Human and Evolutionary Biology Section Department of Biological Sciences University of Southern California Los Angeles CA 90089 USA; ^21^ Department of Anthropology University of Southern California Los Angeles CA 90089 USA; ^22^ School of Kinesiology Faculty of Health Sciences University of Western Ontario 1151 Richmond St London ON N6A 3K7 Canada; ^23^ Canadian Centre for Activity and Aging University of Western Ontario 1201 Western Rd London ON N6G 1H1 Canada; ^24^ Graduate Program in Neuroscience University of Western Ontario 1151 Richmond St London ON N6A 3K7 Canada; ^25^ School of Psychology and Centre for Brain Research University of Auckland Auckland 1030 New Zealand; ^26^ Center for Epidemiological Research in Nutrition and Health Department of Nutrition School of Public Health Universidade de São Paulo São Paulo 05508‐070 Brazil; ^27^ Research Group Degenerative and Chronic Diseases, Movement Faculty of Health Sciences Brandenburg University of Potsdam 14476 Potsdam Germany

**Keywords:** brain Health, cerebrovascular health, effective connectivity, microvascular health, sedentary behavior, young adults

## Abstract

Acknowledging the detrimental effects of prolonged sitting, this study examined the effects of an acute exercise break during prolonged sitting on executive function, cortical hemodynamics, and microvascular status. In this randomized crossover study, 71 college students completed three conditions: (i) uninterrupted sitting (SIT); (ii) SIT with a 15 min moderate‐intensity cycling break (MIC); and (iii) SIT with a 15 min vigorous‐intensity cycling break (VIC). Behavioral outcomes, retinal vessel diameters (central retinal artery equivalents [CRAE], retinal vein equivalents [CRVE], arteriovenous ratio [AVR]), cortical activation, and effective connectivity were evaluated. Linear mixed models identified significant positive effects of exercise conditions on behavioral reaction time (RT), error rate, and inverse efficiency score (*β* = −2.62, −0.19, −3.04: *ps* < 0.05). MIC and VIC conditions produced pre‐to‐post‐intervention increases in CRAE and CRVE (*β* = 4.46, 6.34), frontal activation, and resting‐state and task‐state causal density (*β* = 0.37, 0.06) (*ps* < 0.05) compared to SIT; VIC was more beneficial for executive function and neurobiological parameters. The effect of AVR on average RT was mediated through task‐based causal density (indirect effect: −0.82). Acutely interrupting prolonged sitting improves executive function, microvascular status, and cortical activation and connectivity, with causal density mediating the microvascular‐executive function link.

## Graphical abstract

Interrupting prolonged sitting with a short bout of exercise break can enhance executive function, improve retinal vessel diameters, and increase cortical activation, along with strengthening frontoparietal network connectivity. These findings underscore the potential of physical activity to boost neurocognitive outcomes in sedentary contexts. Additionally, the task‐based causal density is identified as a mediator linking microvascular health and executive function.

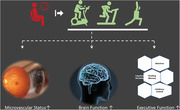

## 1 Introduction

Accumulating evidence suggests that single bouts of physical exercise can transiently improve cognitive performance.^[^ ^1^ ^]^ Since its inception, this research field has shifted its focus from investigating processes such as sensory and perceptual memory to scrutinizing the effects of physical exercise on higher‐order cognition, such as executive function—including inhibitory control, working memory, and cognitive flexibility.^[^ ^1^ ^]^ Regardless of the task used to probe executive functioning, previous studies examining the acute effects of physical exercise on inhibitory control (defined as the ability to resist impulses and distractions) typically reported improved post‐exercise performance.^[^ ^2^ ^]^ Such improvements in inhibitory control processes are relevant to many aspects of everyday living including academic engagement.^[^ ^3^ ^]^ For example, executive functions are closely related to successful school performance because domains of executive functions such as inhibitory control encompass the cognitive mechanisms that organize and regulate goal‐directed behavior in an open environment.^[^ ^4^ ^]^ The Stroop task in general^[^ ^5^ ^]^ and the dual‐task Stroop task in particular are established measures of inhibitory control whereas the latter can also mirror the multitasking demands that can occur in academic and real‐world contexts.^[^ ^6^ ^]^

A considerable portion of the research investigating the relationship between acute physical exercise and cognitive function has been conducted in controlled laboratory settings.^[^ ^7^ ^]^ Although the laboratory environment enables precise measurements and the rigorous control of environmental factors, it may not fully mirror the complex and dynamic real‐life scenarios in which several confounders such as environmental distractions can influence the effects of acute physical exercise on cognitive performance.^[^ ^8^ ^]^ Younger adults, particularly those in educational institutions, often spend extended periods sitting in classrooms or studying, which can present a considerable challenge to maintaining optimal cognitive performance.^[^ ^8^ ^]^ To examine the effects of acute physical exercise on cognitive performance in such an ecologically valid school or university setting, researchers have begun investigating in younger adults (e.g., college students) the effects of acute physical exercise breaks during prolonged sitting on cognitive performance.^[^ ^9^ ^]^ However, this research has yielded mixed results.^[^ ^10^ ^]^ For example, Mullane et al.^[^ ^10a^ ^]^ observed cognitive benefits among nine overweight young adults in response to acute bouts of standing and light‐intensity physical exercise (i.e., cycling and walking) during six hours of prolonged sitting. In contrast, Sperlich et al. observed a non‐significant difference in inhibitory control (assessed by the Stroop task) between two conditions wherein twenty students were required to remain seated for three hours or for the same amount of time while interrupted with a 6 min exercise bout following one hour of sitting.^[^ ^10b^ ^]^ Given the preliminary nature of these findings, factors such as small sample size and methodological heterogeneity may influence the generalizability of the results.

Notably, the neurobiological mechanisms that underlie the cognitive benefits of acute physical exercise breaks during prolonged sitting were poorly understood.^[^ ^7^, ^11^ ^]^ Preliminary evidence from laboratory experiments showed that breaking up prolonged sitting by acute physical exercise may prevent a decline in cognitive performance by increasing endothelial shear stress^[^ ^12^ ^]^ and cerebral blood flow,^[^ ^13^ ^]^ elevating brain neurotrophic factors (e.g., brain‐derived neurotrophic factor),^[^ ^14^ ^]^ and accelerating anti‐inflammatory function.^[^ ^15^ ^]^ Based on a multilevel approach,^[^ ^16^ ^]^ a better understanding of the neurobiological mechanisms that underlie acute physical‐exercise‐induced changes in cognitive performance should not only focus on cellular and molecular mechanisms but should also include the investigation of changes on the functional brain level (e.g., using functional imaging techniques).^[^ ^16^ ^]^ In this context, functional near‐infrared spectroscopy (fNIRS) has emerged as a promising and versatile tool for understanding the effects of acute physical exercise on cognitive performance. The fNIRS is relatively robust against motion‐related artifacts and enables monitoring of functional brain changes (i.e., cortical hemodynamics) with good spatial and temporal resolution.^[^ ^17^ ^]^

In this context, two studies have investigated the effects of acute physical exercise during periods of prolonged sitting on cognitive performance and cortical hemodynamics obtained via fNIRS.^[^ ^18^ ^]^ Yu et al. (2022) investigated the effects of a 15 min acute light‐intensity physical exercise (cycling) break between two 45 min periods of prolonged sitting in 60 college students.^[^ ^18b^ ^]^ Episodic memory performance was not significantly changed by the acute physical exercise break but the nodal efficiency (efficiency of information transfer within brain networks) in the anterior prefrontal cortex during memory encoding, and the nodal efficiency and degree centrality (prominence of a region based on its connection) of the orbitofrontal cortex during memory retrieval were improved.^[^ ^18b^ ^]^ Additionally, during memory encoding, the acute physical exercise break modified functional connectivity between the anterior and dorsolateral prefrontal cortices.^[^ ^18b^ ^]^ Heiland et al. provided evidence that a 3 min break every 30 min during 3 h of prolonged sitting was associated with lower activation of the right prefrontal cortex and improved working memory performance, mood, and alertness in 13 healthy adults.^[^ ^18a^ ^]^ Collectively, these results suggest that acute light‐intensity physical exercise breaks during periods of prolonged sitting can influence cortical hemodynamics, although the effects on behavioral indices may differ between different cognitive domains (e.g., episodic memory, working memory). Notably, however, it is unclear whether physical exercise intensity (e.g., moderate intensity versus vigorous intensity) differentially influences cortical hemodynamics and executive function during periods of prolonged sitting, necessitating further research to better understand potential dose‐response relationships.

To further elucidate potential neurobiological mechanisms that are associated with the acute physical exercise‐induced changes in cortical hemodynamics, we also investigate potential associations between cortical hemodynamics and the microvascular status. Elucidating such potential associations is motivated by the fact that some studies observed significant correlations between indicators of microvascular status and cortical hemodynamics obtained during a handgrip exercise^[^ ^19^ ^]^ and cognitive test.^[^ ^20^ ^]^ In the current study, we utilized retinal vessel analysis, an economical yet effective method, to operationalize the microvascular status of an individual.^[^ ^21^ ^]^ Retinal vessel analysis offers valuable insights into the microvascular status of the brain because cerebral and retinal vessels have similar physiological and morphological properties due to their shared embryological origin.^[^ ^22^ ^]^ Furthermore, cross‐sectional research has examined the relationships between physical fitness (i.e., muscular strength, speed agility, and cardiorespiratory fitness), retinal microcirculation, and cognitive function.^[^ ^23^ ^]^ This cross‐sectional study involving 347 primary school children revealed that indicators of retinal microcirculation independently contributed to variance in cognitive performance, particularly concerning inhibitory control and information processing.^[^ ^24^ ^]^ Additionally, a randomized controlled trial with 35 adolescents, focusing on an 8‐week aerobic and coordinative training program, observed a positive correlation between changes in retinal arteriolar diameter and cognitive performance improvements, specifically in reaction time (RT) during an inhibitory control task.^[^ ^25^ ^]^ Collectively, these findings highlight the potential of retinal vessel diameters as a proxy of the microvascular status to better understand the underlying neurobiological mechanisms that contributed to physical exercise‐induced changes in cognitive performance in general and physical exercise‐induced changes in cortical hemodynamics (i.e., obtained during a cognitive task) in particular. However, potential associations between changes in retinal vessel diameters, cortical hemodynamics, and cognitive performance changes in response to acute physical exercise breaks have not yet been studied.

To address the above‐mentioned knowledge gaps, the current study used a randomized crossover design to investigate the acute effects of physical exercise breaks during two periods of prolonged sitting on executive function (measured via the dual‐task Stroop task), cortical hemodynamics (measured by fNIRS), and retinal microvascular status. To simulate real‐world scenarios, the duration of the two periods of prolonged sitting and the exercise break were set to coincide with two consecutive classes and recess times at Chinese universities. Our primary aims were to 1) investigate among young adults how acute physical exercise breaks of different intensities (vigorous‐ and moderate‐intensity cycling) affect dual‐task Stroop task performance (i.e., baseline, color‐dual task, and lexical‐dual task Stroop performance), cortical hemodynamics (i.e., activation and effective connectivity), and microvascular status following prolonged sitting in an academic setting, and 2) examine the relationships between physical exercise‐induced changes in executive function, cortical hemodynamics, and retinal vessel diameters. Based on the current evidence,^[^ ^18^, ^26^ ^]^ we hypothesized that physical exercise breaks during two periods of prolonged sitting would enhance executive function and cortical hemodynamics. We also hypothesized that interrupting prolonged sitting with acute physical exercise would increase retinal vessel diameters, reflecting enhanced microvascular function.^[^ ^27^ ^]^ Furthermore, based on the literature,^[^ ^7^, ^28^ ^]^ we predicted that vigorous‐intensity exercise cycling would yield greater benefits for both behavioral and neurobiological outcomes. Additionally, we hypothesized that improvements in executive function and microvascular status would be mediated by changes in cortical hemodynamics.

By using measures of different levels of analysis (i.e., retinal microvascular status, cortical hemodynamics, and executive functions) and simulating a realistic academic setting, this study will broaden our knowledge of the effects of acute physical exercise breaks during periods of prolonged sitting and its potential neurobiological mechanisms, on which direct empirical evidence is currently scant in the literature.^[^ ^11^, ^29^ ^]^ Thus, the to‐be‐expected findings of the current study have great potential to inform not only future research work but also public health actions.

## 2 Experimental Section

### 2.1 Participants

College students were recruited through campus poster advertisements. Inclusion criteria were: 1) 18 and 25 years of age;^[^ ^30^ ^]^ 2) normal or corrected‐to‐normal vision;^[^ ^31^ ^]^ 3) right‐handedness indicated by self‐report;^[^ ^32^ ^]^ 4) no history of cardiovascular, metabolic, gastrointestinal, neurological, or psychiatric disorders; and 5) regular menstrual cycles for female participants.^[^ ^33^ ^]^ Exclusion criteria were: 1) any medical conditions requiring ongoing treatment; 2) pregnancy or lactation;^[^ ^34^ ^]^ 3) recent adherence to strict diets (e.g., ketogenic diet);^[^ ^35^ ^]^ and 4) use of substances such as caffeine, alcohol, or nicotine within 48 hours before each trial.^[^ ^36^ ^]^ All participants were able to safely participate in the maximal incremental exercise test and the acute cycling bouts based on the Physical Activity Readiness Questionnaire.^[^ ^37^ ^]^ Each participant signed an informed written consent form before participating. The Ethical Committee of Shenzhen University (No: PN‐2020‐041) approved the research protocol in compliance with the latest version of the ethical principles of the Declaration of Helsinki.

The sample size using the “simr” package in R was calculated,^[^ ^38^ ^]^ simulating a dataset aligned with this study design. The “powerSim” function was employed to run 100 simulations to estimate model power.^[^ ^38^ ^]^ The sample size was iteratively increased until reaching 80% power at a sample size of 63 participants. Ultimately, a total of 71 participants (20.94 ± 1.93 years, 50.70% females) were recruited (Appendix 1).

### 2.2 Study Design

**Figure 1:**
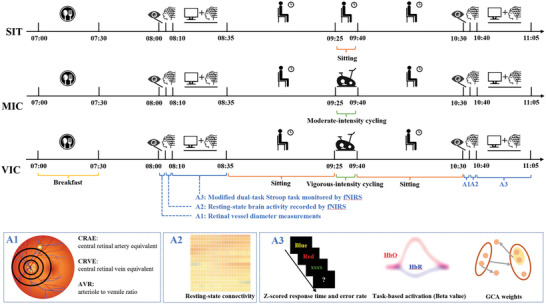
The schematic illustration of the three experimental conditions (i.e., SIT versus MIC versus VIC). (Notes. The primary outcomes of assessments 1–3 were also separately shown at the bottom of the figure; SIT, condition of 115 min uninterrupted sitting [control]; MIC, experimental condition 1 with 15 min moderate‐intensity cycling; VIC, experimental condition 2 with 15 min vigorous‐intensity cycling; fNIRS, functional near‐infrared spectroscopy; A1, assessment 1; A2, assessment 2; A3, assessment 3; CRAE, central retinal artery equivalent; CRVE, central retinal vein equivalent; AVR, arteriole‐to‐venule ratio; HbO, oxyhemoglobin; HbR, deoxyhemoglobin; GCA, Granger causality analysis).

A single‐blind randomized crossover design with pre‐ and post‐intervention comparisons was used. Participants completed each of the following experimental conditions in pseudo‐counterbalanced order with at least a 7‐day washout period between conditions (**Figure** **1**): i) 115 min of uninterrupted sitting [SIT: control]; ii) 50 min of sitting + 15 min of moderate‐intensity cycling + 50 min of sitting [MIC: experimental condition 1]; iii) 50 min of sitting + 15 min of vigorous‐intensity cycling + 50 min of sitting [VIC: experimental condition 2]. To increase ecological validity by simulating the typical class and break durations in most Chinese universities, an acute physical exercise break of 15 min was implemented between two 50 min periods of sitting. Previous work^[^ ^7^, ^39^ ^]^ indicated that breaks within a 10‐20 min range effectively yield cognitive benefits, supporting the selection of this time interval. Participants were randomly assigned to three experimental conditions using R software's random number generator (sample() function).^[^ ^40^ ^]^ A familiarization session was completed approximately three days (±1 day) before the experiment started, during which participants were familiarized with the cognitive assessment and cycling protocols.

In the familiarization session, participants’ sex and age were recorded. Body mass index (BMI) was calculated as body weight in kilograms divided by the square of height in meters, assessed by an automatic portable stadiometer (BSM370, Seoul 135–960, Korea). The Chinese version of the International Physical Activity Questionnaire‐Short Form which has acceptable psychometric properties^[^ ^41^ ^]^ was used to evaluate participants’ moderate‐ and vigorous‐intensity physical activity. As recommended, these activities were weighted by their energy requirements defined in the Metabolic Equivalent of Task (minutes/week) to provide indices for the individual physical activity level.^[^ ^42^ ^]^ The 19‐item Pittsburgh Sleep Quality Index was used to record subjective sleep quality in the past month, in which the global score was derived from 7 components of sleep (subjective sleep quality, sleep latency, sleep duration, sleep efficiency, sleep disturbance, use of sleep medication, and daytime dysfunction).^[^ ^43^ ^]^ The 21‐item Depression, Anxiety, and Stress Scale was used to measure the emotional states of depression, anxiety, and stress, respectively.^[^ ^44^ ^]^ In addition, we used the 8‐item visual analog mood scales to evaluate different mood states, namely feeling afraid, confused, sad, angry, energetic, tired, happy, and tense.^[^ ^45^ ^]^ All the above‐mentioned questionnaires were analyzed according to established recommendations and guidelines.

To account for potential hormonal influences on brain function, all female participants conducted the three experimental trials during the early to mid‐luteal phase of their menstrual cycle.^[^ ^33^ ^]^ During the 48 h before each visit, participants were instructed to refrain from caffeine, alcohol, nicotine, and moderate‐to‐vigorous‐intensity physical activity, and all participants reported adhering to these instructions. Participants were required to consume the same standardized meals (i.e., 55%–58% carbohydrate, 29%–31% fat, and 12%–15% protein) on the day before each visit. The evening before each experimental session, dinner was consumed between 19:00 and 21:00.

### 2.3 Maximal Incremental Exercise Test

The maximal incremental exercise test was conducted seven days before the beginning of the experimental conditions to evaluate participants’ cardiorespiratory fitness level and to set the participant‐specific exercise intensity. After a 3 min warm‐up at 25 W, participants pedaled on an electric‐controlled cycle ergometer (Ergoline Ergoselect 100, Germany) against an initial workload of 50 W, which increased by 25 W every 3 min until volitional exhaustion. The participants were asked to maintain a pedalsling rate of 60 ± 5 revolutions per minute until volitional exhaustion, which was characterized by meeting two or more of the following criteria:^[^ ^46^ ^]^ i) rating of perceived exertion (RPE) ≥ 18; ii) peak heart rate (HR) ≥ 90% of age‐predicted maximum (220 – age); and iii) respiratory exchange ratio ≥ 1.15. Respiratory gases were collected and analyzed using a gas analyzer (Vmax Encore System, CareFusion Corp., United States), which was prepared according to the instructions of the manufacturer. As peak maximal oxygen consumption (VO2_peak_), the average of 15 seconds of the highest oxygen uptake in the final stage was considered.^[^ ^46^ ^]^

### 2.4 Experimental Day Protocol

Participants were instructed to arrive at the laboratory (temperature of 26 °C, humidity of 40%, lighting intensity of 400 lux, background noise below 40 decibels) at ≈07:00 following an overnight fast (>10 h) and had 30 min to consume breakfast. Breakfast remained the same across all the experimental conditions to avoid influences of nutritional composition and energy contribution on cognitive performance.^[^ ^47^ ^]^ Retinal vessel diameter measurements were conducted between 08:00 and 08:05, followed by a 5 min record of resting‐state brain activity by fNIRS. Subsequently, between 8:10 and 8:35, a modified dual‐task Stroop task was completed while cortical hemodynamics were concurrently measured via fNIRS. Next, participants in the SIT condition started 115 min of uninterrupted sitting between 08:35 and 10:30. Participants in MIC and VIC conditions performed 15 min of cycling after the first 50 min sitting period. In line with other studies,^[^ ^14^, ^48^ ^]^ participants were instructed only to leave their chairs if they completed the predetermined cycling exercise, although they were permitted to do so if needed. Participants were allowed to read books/magazines, work on a laptop, do homework, or send email messages during seated rest, and avoid activities that may cause a considerable change in mood (e.g., watching emotional movies).^[^ ^49^ ^]^ The 50 min sitting period was aligned with the duration of class at most Chinese universities. During the experimental procedure, participants were provided with water ad libitum but were not allowed to eat. Lastly, retinal vessel diameters (10:30–10:35), resting‐state brain activity recorded by fNIRS (10:35–10:40), and dual‐task Stroop performance with concurrent fNIRS (10:40–11:05) were re‐evaluated after the period of prolonged sitting (Figure 1).

During the physical exercise break, participants in the MIC and VIC conditions cycled continuously for 15 min at moderate (i.e., ≈60% of VO_2peak_) or vigorous (i.e., ≈80% of VO_2peak_) intensity on a Monark cycle ergometer (839E, Varberg, Sweden), respectively.^[^ ^50^ ^]^ The duration of the physical exercise break was aligned with the regular break duration between two lessons used at most Chinese universities. The exercise‐intensity classification was based on the American College of Sports Medicine's^[^ ^50^ ^]^ definitions for exercise work rates. The preferred saddle height was determined in the familiarization session. The RPE scores^[^ ^51^ ^]^ and HR assessed by a monitor (Polar F4M BLK, Kempele, Finland) were collected at baseline, at 3 min intervals, and immediately after the cessation of the acute bout of physical exercise in MIC and VIC conditions.

### 2.5 Dependent Variables

#### 2.5.1 Retinal Vessel Analysis

In the domains of ophthalmology and vascular health research, three typical indicators, namely the central retinal arteriolar equivalent (CRAE), central retinal venular equivalent (CRVE), and the arteriolar‐to‐venular ratio (AVR), were used to operationalize the microvascular status of an individual.^[^ ^22^ ^]^ Although CRAE and CRVE provide specific insights into the microvascular patterns of arterial and venous microvascular status, respectively, the AVR serves as a broader indicator, reflecting the overall regulatory state of the microcirculation system.^[^ ^23^ ^]^

Retinal vascular diameters of the right eye were assessed using computer‐assisted quantitative assessment software (IVAN, University of Wisconsin, Madison, USA), with images captured by a fundus camera (TRC‐NW400).^[^ ^52^ ^]^ The overlying grid placement, vessel type identification, and vessel width measurement were automatically performed at an angle of 45° with an optic disc at the center.^[^ ^52^ ^]^ Three images were projected at the same magnification, converted to digital images through the scanner, and calibrated based on a standard disc diameter of 1800 mm.^[^ ^52^ ^]^ The six largest arterioles and six largest venules coursing through an area of 0.5‐to‐1.0‐disc diameters from the optic disc margin were measured and used to calculate the average CRAE and CRVE. The calculation of CRAE and CRVE was developed by Hubbard et al. (1999)^[^ ^53^ ^]^ and Knudtson et al. (2003).^[^ ^54^ ^]^ The diameters of retinal arterioles and venules were calculated based on branching coefficients (0.88 for arterioles and 0.95 for venules), which linked parent trunk vessels with branches.^[^ ^54^ ^]^

An iterative procedure was applied to pair the largest vessels with the smallest one and repeat until a single number (central vessel equivalent) was reached.^[^ ^54^ ^]^ The AVR was computed as CRVE divided by CRAE.^[^ ^54^ ^]^ In the current study, trained graders conducted all assessments and analyses of retinal measures. A reproducibility study was undertaken to ensure consistency measurement of CRAE, CRVE, and AVR, with intraclass correlation coefficients ranging from 0.81‐0.86. The assessment followed the standard operating procedures proposed by Streese et al.^[^ ^55^ ^]^

#### 2.5.2 Cognitive Paradigm – the Modified Dual‐Task Stroop Task

**Figure 2:**
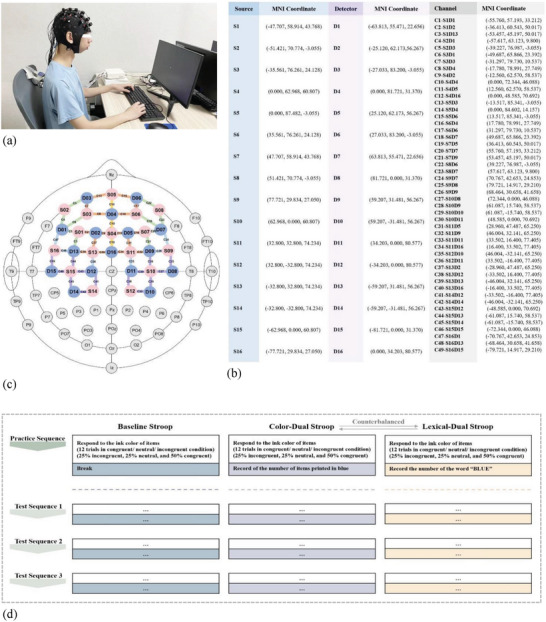
a,b) Visualization of the functional near‐infrared spectroscopy setup and recording, c) the spatial registration of the fNIRS channels, and d) the test procedures of the modified dual Stroop task. (Notes. Figure 2a, equipment worn in the actual experimental environment; Figure 2b, coordinates of sources, detectors, and channels; Figure 2c, location of sources and detectors; Figure 2d, modified dual‐task Stroop task paradigm. In Figure 2c, the pink circle represents the source, while the blue circle represents the detector. In Figure 2c, the yellow, orange, green, blue, and purple lines separately represent channels in the medial frontal [C10, C12, C14], superior frontal [C2, C7‐9, C11, C13, C15‐17, C19, C31‐32, C34, C37, C39‐40], middle frontal [C1, C3‐6, C18, C20‐23], precentral [C24, C26, C33, C38, C47‐48], and parietal [C25, C27‐30, C35, C36, C41‐46, C49] regions. In Figure 2d, three blocks of modified dual‐task Stroop task were well represented: the baseline Stroop block ran first, and then the Color‐Dual Stroop block and the Lexical‐Dual block ran in a counterbalanced order. Each block comprised a practice sequence and 3 test sequences, with 12 trials in each sequence.). (Abbreviations. S, source; D, detector; C, channel).

A modified dual‐task Stroop task, which was originally developed by Ward et al.,^[^ ^6^ ^]^ was used (see **Figure** **2**). The dual‐task Stroop task consisted of three blocks conducted in the following order: i) the baseline Stroop block, ii) the color‐dual Stroop block, and (iii) the lexical‐dual Stroop block. As shown in Figure 2, the order of the latter two blocks was counterbalanced. Each block comprised a practice sequence and three test sequences, with 12 trials in each sequence. There were 25% incongruent stimuli (i.e., the word “BLUE,” “GREEN,” and “YELLOW” printed in an incongruent color), 25% neutral stimuli (i.e., groups of Xs [XXXX] printed in blue, green, or yellow), and 50% congruent stimuli (i.e., the word “BLUE,” “GREEN,” “YELLOW” printed in a congruent color) trials in one block. For each trial, a central fixation was first presented for 8000 ms. A stimulus remained on the screen for 3000 ms, and an interstimulus interval of 500 ms was employed. Each block lasted ≈504 s.

In the baseline Stroop condition, participants were instructed to indicate the ink color by pressing the colored keys on the keyboard with the right hand as quickly and accurately as possible. In the color‐dual task Stroop condition, participants continued responding to the ink color of stimuli but also needed to keep track of the number of items printed in blue and enter this number via the keyboard when prompted to do so. In the lexical‐dual task Stroop condition, participants were instructed to indicate the ink color of the items while keeping in mind the number of times they saw the word “BLUE,” regardless of the ink color. The count of blue items or the word “BLUE” was restarted in each sequence. Participants were seated ≈50 cm from the monitor during the whole task. The inverse efficiency score (IES) was calculated as follows: IES = mean RT for correct response/(1‐proportion of decision errors), with a higher value indicating lower efficiency (more time taken and/or more errors). As recommended by Ward et al.,^[^ ^6^ ^]^ the z‐scored RT, error rate (ER), and IES were calculated for normalization, facilitation of more robust statistical analysis, and combination of different measures.

#### 2.5.3 fNIRS Data Acquisition and Quality Check

The fNIRS data were collected from the NIRSport2 device (NIRx Medical Technologies, LLC) operating in the continuous‐wave mode (Figure 2). The optodes were set in an fNIRS‐EEG compatible cap using the international 10‐10 system for EEG electrode placement. In the current study, the fNIRS‐montage included 16 LED sources and 16 avalanche photodiode detectors with an inter‐optode distance of ≈3 cm, which covered nine regions of interests (ROIs;, i.e., medial frontal [C10, C12, C14], left superior frontal [C2, C7, C8, C9, C13, C37, C39, C40], left middle frontal [C1, C3, C4, C5, C6], left precentral [C38, C47, C48], left parietal [C41, C42, C43, C44, C45, C46, C49], right superior frontal [C11, C15, C16, C17, C19, C31, C32, C34], right middle frontal [C18, C20, C21, C22, C23], right precentral [C24, C26, C33], and right parietal [C25, C27, C28, C29, C30, C35, C36] regions). The anatomical locations of sources, detectors, and 46 channels were estimated and registered to the Montreal Neurological Institute probabilistic reference system using a Patriot 3D Digitizer (Polhemus, Co., USA).^[^ ^56^ ^]^ The wavelengths of light emitted by the source were 760 nm and 850 nm. The data were recorded continuously at a sampling rate of 10.2 Hz using the NIRx acquisition software Aurora fNIRS. The quality check and segmentation of fNIRS data were conducted before the further processing of the data, and channels with a bad signal quality were excluded from subsequent analyses (i.e., channels with a coefficient of variation equal to or higher than 15%).^[^ ^57^ ^]^

#### 2.5.4 fNIRS Data Analysis: Preprocessing Pipeline and Task‐Based Activation

Regarding the fNIRS data analysis, it followed established guidelines and recommendations.^[^ ^17^, ^58^ ^]^ In brief, task‐based fNIRS data in the three blocks (i.e., baseline Stroop block versus color‐dual Stroop block versus lexical‐dual Stroop block) were analyzed using the NIRS Brain AnalyzIR Toolbox.^[^ ^59^ ^]^ During preprocessing, raw light intensity (pre‐block time: 15 s; block time: 504 s) was loaded and converted to optical density and then to oxygenated (HbO) and deoxygenated (HbR) hemoglobin concentration using the Modified Beer‐Lawbert Law.^[^ ^60^ ^]^ At the first level (participant level) of statistics, a general linear regression analysis was performed with an autoregressive iterative reweighted least‐squares approach, which corrected the serially correlated errors with pre‐whitening and down‐weighted outliers caused by motion artifacts by robust weighted regression. A spatial principal component filter was applied to correct artifacts arising from motion and systemic physiology.^[^ ^59^ ^]^ A canonical hemodynamic response function model was used in the sensitivity‐specificity analysis. At the second level (group level) of statistics, the group mean for each condition was calculated. Each condition's estimated regression coefficients (i.e., beta value) represented the estimated strength of hemodynamic activity. False discovery rate (FDR) correction was used to conceptualize the rate of type I errors in multiple comparisons.

#### 2.5.5 fNIRS Data Analysis: Preprocessing Pipeline and Effective Connectivity Analysis

The 5 min HbO time‐series data before the modified dual‐task Stroop task^[^ ^61^ ^]^ was used for the effective connectivity analysis (Granger causality analysis, GCA) of resting state,^[^ ^62^ ^]^ as it previously showed maximum agreement with fMRI resting‐state signals.^[^ ^63^ ^]^ The GCA was used to determine whether one time series may predict another, revealing probable causal relationships between variables in time‐dependent data.^[^ ^62^ ^]^ Effective connectivity analyses were also conducted for the HbO time series data in the three blocks (baseline, color‐dual, and lexical‐dual Stroop blocks) and during the whole task. A band‐pass filter between 0.01 and 0.08 Hz^[^ ^64^ ^]^ was used before converting the optical density data to HbO time series data using the modified Beers‐Lambert law (differential path‐length factor = 6). Moreover, the first few components acquired from the principal components analysis were regressed out to reduce the systemic physiological noises.^[^ ^65^ ^]^

Using preprocessed HbO time‐series data, vector autoregressive (VAR) models were developed utilizing the MATLAB Multivariate Granger Causality toolbox (version 1; MathWorks, Natick, MA, USA) to estimate causal interactions across ROIs.^[^ ^62a^ ^]^ The optimal model order was selected according to the Bayesian Information Criterion (BIC) due to its balance between model fit and complexity.^[^ ^62a^ ^]^ Model stability was achieved by ensuring all eigenvalues lay inside the unit circle.^[^ ^62a^ ^]^ The model's goodness‐of‐fit was verified using the Akaike Information Criterion (AIC) alongside residual white noise tests.^[^ ^62a^ ^]^ Maximum likelihood estimation was used in parameter estimation of the VAR model for efficiency and consistency in the context of multivariate time‐series data.^[^ ^62a^ ^]^ The *F*‐statistic quantified the extent to which each lagged value contributed to the prediction of future values of the dependent variable.^[^ ^62a^ ^]^ Causal density served as a comprehensive metric for assessing the strength and distribution of causal interactions within the cortical network.^[^ ^62a^ ^]^ Statistical significance was set at *p* < 0.05, and the *p*‐values of the multiple comparisons in hypothesis testing were corrected using FDR estimation.^[^ ^62a^ ^]^ To provide a more comprehensive view of hemodynamic responses, we also conducted effective connectivity analyses for HbR.

### 2.6 Statistical Analyses

Linear mixed models (LMMs) were used to investigate the effects of exercise breaks on behavioral outcomes, such as RT, ER, and IES, across the different experimental conditions (i.e., SIT versus MIC versus VIC), Stroop conditions (baseline Stroop versus color‐dual Stroop versus lexical‐dual Stroop), and assessment times (preintervention versus postintervention). Our models included interactions between factors and controlled for sex, age, and BMI.^[^ ^66^ ^]^ The random effect of participants was incorporated to account for intra‐subject variability.^[^ ^67^ ^]^ Similarly, for task‐based fNIRS data, models were built on beta values of each ROI and causal density. In the models of retinal vessel diameter measurements (i.e., CRAE, CRVE, and AVR) and causal density of resting‐state fNIRS data, the experimental condition and assessment time were regarded as fixed effects, while the random intercept for each participant was set to account for within‐participant correlations. The AIC and BIC balanced model fit and complexity.^[^ ^66^ ^]^ Maximum likelihood estimation was used for fixed and random effect parameter estimation.^[^ ^66^ ^]^ Effect sizes are reported as beta values (β) from the LMM, which represent the estimated change in the outcome variable per unit change in the predictor, adjusted for fixed and random effects.^[^ ^68^ ^]^ Post‐hoc statistical analyses were performed to decompose interaction effects and examine simple effects at different levels of factors (i.e., experimental condition, Stroop condition, and assessment time points) using estimated marginal means.^[^ ^66^ ^]^ To ensure the validity of LMMs, normality was assessed via Q‐Q plots and the Shapiro‐Wilk test.^[^ ^69^ ^]^ Homogeneity of variance was evaluated using residual versus fitted plots and the Breusch‐Pagan test,^[^ ^70^ ^]^ and independence was tested using the Durbin‐Watson test for repeated measures data.^[^ ^71^ ^]^ If normality or homoscedasticity assumptions were violated, variance‐stabilizing transformations were applied.^[^ ^72^ ^]^ For cases where assumptions were difficult to meet or fully resolve, bootstrapping was used to ensure robust estimates.^[^ ^73^ ^]^ Corresponding analyses were conducted using “lme4”^[^ ^74^ ^]^ and “emmeans”^[^ ^75^ ^]^ packages of R software (version 3.3.3; The R Foundation, Vienna, Austria).

Mediation analysis was performed to investigate whether specific measures of cortical activity (i.e., the average change of beta values of HbO, the causal density of resting state, and during the whole task from pre‐intervention to post‐intervention) mediated the potential relationship between microvascular parameters obtained via retinal vessel analysis (i.e., the average changes of CRAE, CRVE, and AVR) and behavioral cognitive performance (the average changes of RT, ER, and IES). In the first step, an explorative correlation analysis to identify variables with statistically significant associations was performed. Afterward, standard mediation models were built through the Mediation Toolbox designed for neuroimaging statistics (Tor Wager's group; https://github.com/canlab/MediationToolbox)^[^ ^76^ ^]^ to check whether mediation effects occur if it was observed statistically significant correlations between specific variables. Confounding variables (i.e., sex, age, and BMI) were included as regressors in the mediation model. Bootstrap‐based significance testing with 10 000 random samples was utilized for statistical mediation analysis. A statistically significant mediation effect was defined as the bias‐corrected bootstrap confidence limits for the indirect path that did not include zero.^[^ ^77^ ^]^ A two‐tailed *p*‐value less than 0.05 was considered statistically significant. The FDR approach was used to control the rate of type I errors in the presence of multiple comparisons.

## 3 Results

### 3.1 Participant’s Characteristics

Participants’ demographics stratified by biological sex are reported in Appendix 1. There were 35 males (age: 20.34 ± 1.81years; BMI: 23.00 ± 4.28 kg m^2^; VO_2peak_: 32.33 ± 7.66 ml kg min^−1^) and 36 females (age: 21.53 ± 2.01 years; BMI: 20.36 ± 3.05 kg m^2^; VO_2peak_: 27.11 ± 5.73 ml kg min^−1^) recruited into the study. Information on mental health, sleep, and regular physical activity levels is also available in Appendix 1. No significant differences in nutrient intake (protein, carbohydrate, and fat) were observed among SIT, MIC, and VIC conditions (*ps* > 0.05) (Appendix 1). For the MIC condition, RPE scores (0–10 and 6–20 scales) and HR (beats per minute) were as follows: baseline (1.23 ± 0.55, 10.00 ± 0.83, 106.01 ± 9.37), 3 min (2.37 ± 0.57, 11.82 ± 1.05, 119.18 ± 8.32), 6 min (3.04 ± 0.66, 12.58 ± 1.09, 128.62 ± 7.03), 9 min (4.56 ± 0.81, 12.83 ± 1.13, 130.20 ± 6.21), 12 min (4.76 ± 0.84, 13.10 ± 1.24, 130.25 ± 5.94), and immediately post‐exercise (4.90 ± 0.94, 13.31 ± 1.34, 131.35 ± 5.35). In the VIC condition, RPE scores (0–10 and 6–20 scales) and HR were similarly reported at each time point: baseline (2.10 ± 0.83, 10.62 ± 1.30, 113.37 ± 12.57), 3 min (3.71 ± 0.68, 12.56 ± 1.53, 135.82 ± 11.07), 6 min (4.61 ± 0.87, 14.52 ± 1.40, 152.49 ± 10.44), 9 min (6.23 ± 0.99, 15.32 ± 1.32, 159.59 ± 9.00), 12 min (6.66 ± 1.23, 15.92± 1.79, 161.63 ± 7.89), and immediately post‐exercise (7.15 ± 1.28, 16.62 ± 1.82, 166.06 ± 8.75).

### 3.2 Linear Mixed Models of Behavioral Cognitive Outcomes (Reaction Time, Error Rate, and Inverse Efficiency Score)

**Figure 3:**
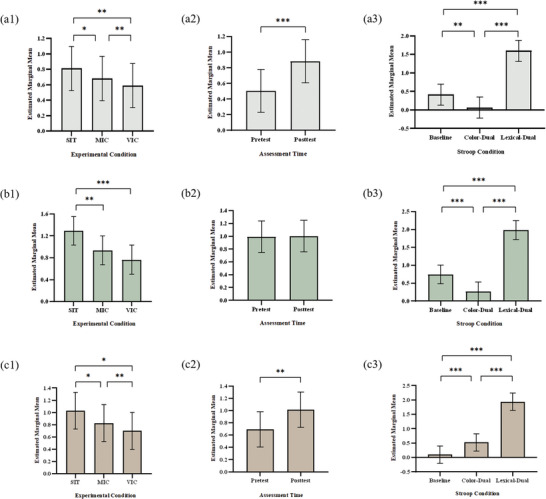
Results of behavioral assessments. (Notes. a1, comparisons of experimental conditions [SIT, MIC, VIC] of reaction time; a2, comparisons of assessment timepoints [pretest, posttest] of reaction time; a3, comparisons of Stroop conditions [baseline, color‐dual, and lexical‐dual Stroop] of reaction time; b1, comparisons of experimental conditions of error rate; b2, comparisons of assessment timepoints of error rate; b3, comparisons of Stroop conditions of error rate; c1, comparisons of experimental conditions of inverse efficiency score; c2, comparisons of assessment timepoints of inverse efficiency score; c3, comparisons of Stroop conditions of inverse efficiency score. Data are shown as the mean and 95% confidence intervals. Statistical comparisons were conducted using estimated marginal means with post‐hoc comparisons of linear mixed models. Asterisks indicate significance levels: *p < 0.05, **p < 0.01.). (Abbreviations. SIT, uninterrupted sitting; MIC, moderate‐intensity cycling; VIC, vigorous‐intensity cycling).

The LMM analysis identified significant main effects of experimental condition, Stroop condition, and assessment time on behavioral outcomes (**Figure** **3** and Appendix 2). The interactions between the experimental condition and assessment time as well as between the experimental condition and Stroop condition were statistically significant for RT (*β* = −1.52; *β* = −1.15) and IES (*β* = −1.77; *β* = −1.40) (*ps* < 0.05). Consistent with our hypothesis, we found that MIC reduced RT, ER, and IES more than SIT from pre‐intervention to post‐intervention (difference in estimated marginal means [emmean]: −0.13, −0.36, −0.20, respectively, *ps* < 0.05). Compared to MIC, VIC showed further reductions in RT, ER, and IES from pre‐intervention to post‐intervention (emmean difference: −0.09, −0.17, −0.13, respectively, *ps* < 0.05). Comparisons between Stroop conditions showed that both color‐dual and lexical‐dual Stroop blocks resulted in a higher level of IES than the baseline Stroop block across experimental conditions (emmean differences: 0.425, 1.832; *ps* < 0.05).

### 3.3 Linear Mixed Models of Retinal Vessel Diameters

Significant interactions between experimental conditions and assessment time were observed for CRAE (*β* = 4.46, *p* < 0.05) and CRVE (*β* = 6.34, *p* < 0.05) (Appendix 3). Compared to SIT, both MIC and VIC significantly increase the CRAE (emmean difference: 0.27, 3.03) and CRVE (emmean difference: 1.14, 3.72) from pre‐intervention to post‐intervention (*ps* < 0.05), which is consistent with our hypothesis. The VIC further significantly increased the CRVE in comparison with MIC from pre‐intervention to post‐intervention (emmean difference: 2.58; *p* < 0.05). The AVR was significantly influenced by both experimental conditions (*β* = 0.0003, *p* < 0.05) and assessment time (*β* = −0.01, *p* < 0.05), but not the interaction between them (*p* > 0.05).

### 3.4 Linear Mixed Models of Beta Values of Cortical Activation

For cortical activation (i.e., the beta value of HbO) (Appendix 4), significant interactions between experimental conditions and assessment time as well as interactions between experimental conditions and Stroop condition were observed for ROI 2 (*β* = 0.08; *β* = 0.10), ROI 3 (*β* = 0.06; *β* = 0.08), ROI 6 (*β* = 0.04; *β* = 0.06), and ROI 7 (*β* = 0.04; *β* = 0.06) (*ps* < 0.05). Compared to SIT, both MIC and VIC induced higher levels of cortical activation in ROI 2 (emmean differences: 0.002, 0.004), ROI 3 (emmean differences: 0.002, 0.005), ROI 6 (emmean differences: 0.001, 0.006), and ROI 7 (emmean differences: 0.002, 0.009) from pre‐intervention to post‐intervention (*ps* < 0.05). The VIC induced a higher level of cortical activation than MIC in these ROIs from pre‐intervention to post‐intervention (emmean difference in ROI 2, 3, 6, and 7: 0.002, 0.003, 0.005, 0.007; *ps* < 0.05).

For hemodynamic response in HbR (i.e., beta value) (Appendix 5), there were no significant interactions between experimental conditions and assessment time or interactions between experimental conditions and Stroop condition observed in the ROIs (*p* > 0.05).

### 3.5 Linear Mixed Models of Resting‐State Causal Density as Well as Granger Causality Analyses

For the resting‐state causal density of HbO, significant effects for experimental condition (*β* = 0.64, *p* < 0.05) and assessment time (*β* = −0.64, *p* < 0.05), as well as their interaction (*β* = 0.37, *p* < 0.05) were observed (Appendix 6). Compared to SIT, both MIC and VIC significantly increased the causal density from preintervention to postintervention (emmean differences = 0.17, 0.17; *ps* < 0.05). For the resting‐state causal density of HbR, no significant main effect (i.e., experiment condition, assessment time, and Stroop condition) or interaction was observed (*p* > 0.05) (Appendix 7). Details of Granger's causal analysis of resting‐state causal density (HbO and HbR) are provided in Appendix 8.

### 3.6 Linear Mixed Models of Task‐Based Causal Density as Well as Granger Causality Analyses

**Figure 4:**
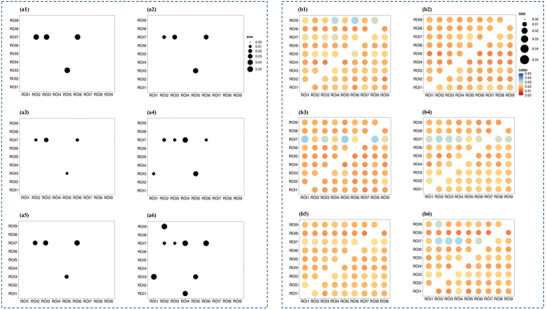
Granger causality analysis of the task‐based causal density of oxyhemoglobin. (Notes. Figure a1‐a6, p values of Granger causality analyses [a1‐2, pre‐intervention and post‐intervention of SIT; a3‐4, pre‐intervention and post‐intervention of MIC; a5‐6, pre‐intervention and post‐intervention of VIC. The black dot indicated the existence of a connectome between two ROIs [from the X axis to the Y axis], with the dot size indicating the value of *p* value. Figure b1‐6 and F‐statistics of Granger causality analyses [b1‐2, pre‐intervention and post‐intervention of SIT; b3‐4, pre‐intervention and post‐intervention of MIC; b5‐6, pre‐intervention and postintervention of VIC]. The color [from red to blue] and size [from the smallest to the largest] of dots indicated the values of F‐statistics [from 0.00 to 0.05]. ROI 1, medial frontal region; ROI 2, left superior frontal region; ROI 3, left middle frontal region; ROI 4, left precentral region; ROI 5, left parietal region; ROI 6, right superior frontal region; ROI 7, right middle frontal region; ROI 8, right precentral region; ROI 9, right parietal region; ROI, region of interest; SIT, condition of 115 min uninterrupted sitting [control]; MIC, experimental condition 1 with 15 min moderate‐intensity cycling; VIC, experimental condition 2 with 15 min vigorous‐intensity cycling.]

**Figure 5:**
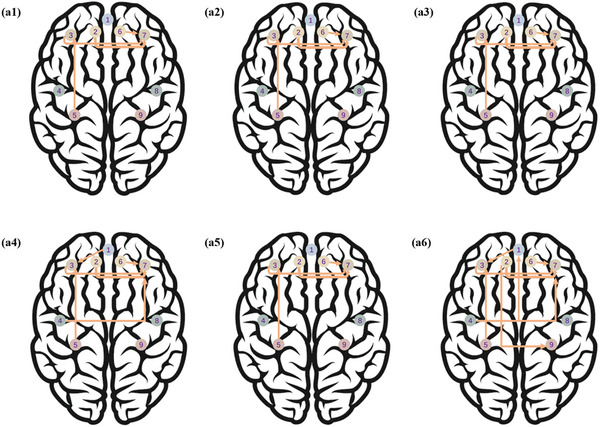
Directional connectomes among the brain regions of interest. (Notes. The directional connectomes among the brain regions of interest were acquired from Granger causality analysis for task‐based causal density of oxyhemoglobin. a1‐2, connectomes of pre‐intervention and post‐intervention of SIT; a3‐4, connectomes of pre‐intervention and post‐intervention of MIC; connectomes of pre‐intervention and post‐intervention of VIC. ROI 1, medial frontal region; ROI 2, left superior frontal region; ROI 3, left middle frontal region; ROI 4, left precentral region; ROI 5, left parietal region; ROI 6, right superior frontal region; ROI 7, right middle frontal region; ROI 8, right precentral region; ROI 9, right parietal region.); ROI, region of interest; SIT, condition of 115 min uninterrupted sitting [control]; MIC, experimental condition 1 with 15 min moderate‐intensity cycling; VIC, experimental condition 2 with 15 min vigorous‐intensity cycling).

For task‐based causal density, significant interactions were observed between experimental condition and assessment time (*β* = 0.06), between experimental condition and Stroop condition (*β* = 0.09), and between assessment time and Stroop condition (*β* = 0.27) (*ps* < 0.05). Compared to SIT, both MIC and VIC enhanced the task‐based causal density from pre‐intervention to post‐intervention (emmean differences: 0.004, 0.007; *ps* < 0.05), which was consistent with our hypothesis. The VIC resulted in a higher level of causal density than MIC from pre‐intervention to post‐intervention (emmean difference: 0.003; *p* < 0.05). Compared to SIT, the MIC induced the connectomes from ROI 1 to ROI 3 and from ROI 4 to ROI 7 from pre‐intervention to post‐intervention (differences in *F* statistics: 0.006, 0.01) (*ps* < 0.05) (**Figures** **4** and **5**). Compared to SIT, the VIC induced the connectomes from ROI 1 to ROI 3, from ROI 2 to ROI 9, from ROI 4 to ROI 1, and from ROI 4 to ROI 7 from pre‐intervention to post‐intervention (differences in *F* statistics: 0.007, 0.01, 0.01, 0.01; *ps* < 0.05) (Figures 4 and 5).

In the LMM of the task‐based causal density of HbR, the experimental condition (*β* = 0.01, *p* < 0.05) but not assessment time or Stroop condition showed significant effects (*ps* > 0.05) (Appendix 10).

### 3.7 Standard Mediation Model

**Figure 6:**
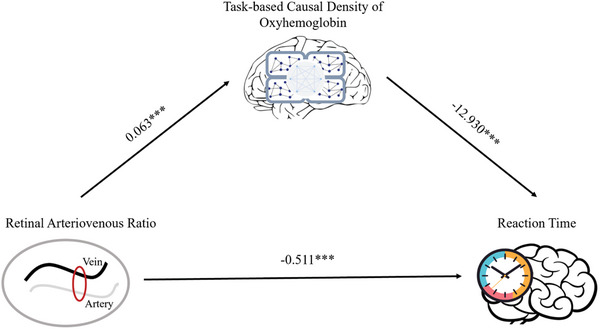
Mediation model. (Notes. Independent variable, retinal arteriovenous ratio; dependent variable, average reaction time; mediator, task‐based causal density of oxyhemoglobin).

Partial correlation analyses among measures are available in Appendix 11. A standard mediation model was built to investigate the effects of AVR on RT through the task‐based causal density of HbO (Appendix 11 and **Figure** **6**). The total effect of AVR on RT was significant (*β* = −1.33, 95%CI [−1.44, −1.22], t = −23.54, *p* < 0.05). When controlling for task‐based causal density of HbO, the direct effect of AVR on RT remained significant (*β* = −0.51, 95%CI [−0.72, −0.30], t = −4.89, *p* < 0.05). The mediation effect was significant, as indicated by the indirect effect of −0.82 (95%CI [−1.00, −0.29]).

## 4 Discussion

The findings supported our hypotheses that acute physical exercise enhanced microvascular status and measures of executive function, and that these effects were mediated by improvements in cortical hemodynamics. We observed a significant improvement in executive function following acute physical exercise breaks, particularly for vigorous‐intensity cycling, which showed superior effects compared to moderate‐intensity cycling. We also noted significant increases in retinal vessel diameters (i.e., CRAE, CRVE, and AVR) and cortical activation in regions associated with executive function. A single bout of physical exercise breaking up prolonged sitting with either moderate or vigorous intensity acutely enhanced both resting‐state and task‐based effective connectivity and causal density of the frontoparietal network. Moreover, findings from the mediation analysis suggested that changes in retinal microvascular status (i.e., AVR) may influence performance via alterations in the causal density of the frontoparietal network during a dual‐task Stroop task.

### 4.1 Influence of An Acute Physical Exercise Break on Executive Function

In terms of behavior, results showed that among college students, an acute physical exercise break during prolonged sitting led to significant improvements on a dual‐task Stroop task, evidenced by shorter RT, lower ER, and reduced IES. These findings support and extend prior research emphasizing that brief periods of physical exercise benefit executive function, specifically inhibitory control, which is essential for controlling impulsive behavior, sustaining concentration, and executing goal‐directed actions.^[^ ^1^, ^2^ ^]^ Moreover, our study adds to previous research as it suggests that acute physical exercise breaks at vigorous intensity resulted in more pronounced improvements in executive function compared to acute physical exercise breaks at moderate intensity. Notably, recent research indicates that implementing vigorous‐intensity exercise breaks in practical settings, such as educational environments, is feasible.^[^ ^78^ ^]^ However, moderate‐intensity exercise is potentially more acceptable to students and could foster long‐term adherence by offering a more pleasurable experience.^[^ ^79^ ^]^ Further, young adults exhibited slower RT and higher IES during sedentary periods, underscoring the negative consequences of prolonged sitting on cognitive function. These findings corroborate existing literature demonstrating the detrimental effects of sedentary behavior on measures of cognitive performance.^[^ ^9^, ^80^ ^]^ Additionally, our study utilized a modified dual‐task Stroop task to assess executive function performance in different Stroop conditions. Across experimental conditions, we found that young adults exhibited poorer cognitive performance in both the color‐dual and lexical‐dual Stroop conditions compared to the baseline single‐task Stroop condition, with the lexical‐dual condition showing the poorest performance. This observation is perhaps related to the fact that the traditional Stroop test probes inhibitory control, while the dual‐task Stroop conditions assessed a broader spectrum of executive functions, including working memory, divided attention, and cognitive flexibility.^[^ ^81^ ^]^ These findings suggest that the increased cognitive load in dual‐task conditions can significantly influence the ability to manage and integrate multiple cognitive processes simultaneously.^[^ ^81^ ^]^ Overall, our results underscore the significant impact of acute physical exercise breaks on multiple aspects of executive performance and underline the potential advantages of integrating acute physical exercise breaks into practical settings, that are characterized by a high level of sedentary behavior (e.g., university classes), to alleviate the detrimental consequences of prolonged sitting on cognitive function.

### 4.2 Influence of An Acute Physical Exercise Break on the Retinal Microvasculature

Our findings suggest that engaging in acute physical exercise breaks during prolonged sitting led to significant increases in retinal vessel diameters, as indicated by variations in CRAE, CRVE, and AVR. Whereas prolonged sitting was associated with decreases in retinal vessel diameters across all measures (CRAE, CRVE, and AVR). These findings align with existing literature demonstrating the beneficial effects of physical activity on microvascular health operationalized via retinal vessel parameters.^[^ ^82^ ^]^ Notably, the vigorous‐intensity exercise break condition resulted in greater changes in vessel diameter metrics compared to the moderate‐intensity condition. This is consistent with Nussbaumer et al.,^[^ ^27a^ ^]^ who found that physical exercises with a maximal effort triggered a more pronounced vasodilatory response than submaximal efforts. This enhanced response likely stems from the stronger activation of autoregulatory mechanisms, such as the myogenic response, during more strenuous activities.^[^ ^27a^ ^]^ These mechanisms can help to maintain adequate blood flow and protect microvascular structures during the elevated blood pressure states induced by strenuous exercise.^[^ ^27a^ ^]^

### 4.3 The Influence of An Acute Physical Exercise Break on Cortical Hemodynamics

Compared to prolonged sitting (SIT), acute physical exercise breaks (both MIC and VIC) were associated with increased activation within the frontal cortex, specifically in the left and right superior frontal and middle frontal regions, across all three blocks of the modified dual‐task Stroop task. Our findings also demonstrate that enhanced cortical activation, particularly in the prefrontal cortex, correlates with improved Stroop performance, indicating a significant relationship between brain activity and cognitive performance during exercise breaks during prolonged sitting.^[^ ^17^, ^83^ ^]^ This suggests that periods of exercise break may enhance neural efficiency, enabling better cognitive performance with less exertion.^[^ ^84^ ^]^ Furthermore, physical exercise breaks also influenced the resting‐state and task‐based causal density within an established frontoparietal executive function network.^[^ ^85^ ^]^ This indicates that acute physical exercise breaks may facilitate efficient recruitment and resource synchronization within and between the local neural structures supporting executive function.^[^ ^86^ ^]^ In the MIC condition, an enhanced connection was observed between ROI 1 (medial frontal region) and ROI 3 (left middle frontal region), and between ROI 4 (left precentral region) and ROI 7 (right middle frontal region). These connections suggest that moderate‐intensity physical exercise breaks promote stronger functional coupling between key frontal regions implicated in executive control processes and motor coordination, potentially facilitating more efficient cognitive and motor performance.^[^ ^86^ ^]^ Besides the effective connectivity observed in the MIC condition, the VIC condition exhibited broader enhancements in connectivity from ROI 2 (left superior frontal region) to ROI 9 (right parietal region), implicating more widespread effects on brain network organization. The acute physical exercise breaks at vigorous intensity likely exert a stronger influence on neural connections, potentially due to increased physiological demands and activation of extra brain areas involved in motor planning, attention, and sensorimotor integration.^[^ ^87^ ^]^ Integrating indicators such as cortical activation, causal density, and effective connectivity provides a comprehensive view of how acute physical exercise breaks can influence cortical hemodynamics in specific brain areas that have been documented to be essential for executive function (e.g., prefrontal cortex).

### 4.4 The Association Between Acute Physical Exercise‐Induced Changes in Retinal Microvasculature, Cortical Hemodynamics, and Cognitive Performance

The results of the mediation analysis suggest that the association between AVR and RT was partially mediated by task‐based causal density in the frontoparietal network. In the present study, AVR as a proxy for microvascular health, was used as an independent variable – specifically referring to post‐to‐pre changes, while task‐based causal density in the frontoparietal network (reflecting cortical hemodynamics) was selected as a mediator, serving as a key intermediary that transmits the influence of microvascular health on cognitive performance.^[^ ^88^ ^]^ The task‐based causal density of the frontoparietal network acts as an indicator of neural activity and functional connectivity, demonstrating the brain's capacity to allocate resources and coordinate activity across cortical regions.^[^ ^89^ ^]^ As hypothesized in previous work,^[^ ^88^ ^]^ our observation that changes in cortical hemodynamics and neural processing efficiency mediate the relationships between microvascular health status (i.e., operationalized by retinal parameter) and cognitive performance, support the notion that microvascular change is a precursor and driver of functional brain change.^[^ ^88^ ^]^ Of note, a correlation between microvascular status and functional brain changes is physiologically plausible but future research is needed to elucidate this phenomenon in more detail and to verify our observations. While this mediation provides evidence for a putative mechanism of acute physical exercise break on cognitive performance, it is also crucial to consider other neurobiological pathways (e.g., changes in neuromodulatory systems such as noradrenergic or dopaminergic systems)^[^ ^27^, ^90^ ^]^ that might explain additional variance of the post‐exercise cognitive performance improvements. Thus, it is recommended that future research investigates the specific neurobiological mechanisms driving the positive effects of acute physical exercise breaks on cognitive performance by considering additional variables such as pupil size (as a proxy for the activity of the noradrenergic system)^[^ ^27b^ ^]^ and spontaneous eye blink rate (as a proxy for the dopaminergic system).^[^ ^90^ ^]^

### 4.5 Limitations, Strengths, and Novelties

It is important to acknowledge several limitations of the current research. First, the generalizability of our findings may be limited due to the specific characteristics of our sample, which consisted solely of college students. Future research should replicate these findings in other cohorts with different ages, physical fitness, or health status to investigate the generalizability of our findings.^[^ ^27a^ ^]^ Second, while our study employed advanced measurement techniques such as fNIRS and retinal vessel analysis, these methods have inherent limitations in terms of spatial resolution, accuracy, and indirect assessment, which should be considered when interpreting the results. Of note, the fNIRS does not enable the detection of activity in subcortical structures and thus cannot provide a complete picture of brain activation patterns. Third, we specifically studied the cortical hemodynamics and microvascular status related to several aspects of executive function; thus, the potential contributions of other neurobiological mechanisms, such as molecular and cellular signaling (e.g., noradrenergic and dopaminergic system), remain elusive and should be considered in future studies. Fourth, while this study focuses on and probes executive function with the modified dual‐task Stroop task, other cognitive domains, such as aspects of attention and memory,^[^ ^91^ ^]^ may also be influenced by acute physical exercise breaks. Thus, future research should consider assessing a broader set of cognitive processes to substantiate the generalizability of these findings. Fifth, this study focuses on the acute effects of physical exercise breaks on executive performance, without assessing the long‐term implications of regular physical exercise breaks on cognitive performance. Based on our findings, it is a promising area for future research to investigate whether implementing acute physical exercise breaks over longer periods (e.g., a semester) can yield lasting cognitive benefits. Sixth, this study assessed cognitive performance following prolonged sitting, without introducing continuous cognitive load (e.g., arising from studying or computer work) during sedentary time. In the real‐world academic settings, individuals often experience continuous cognitive demands during sitting (e.g., listening to the lecture while taking notes).^[^ ^92^ ^]^ Thus, to better mimic practical settings, future research should investigate how inducing different levels of cognitive load during periods of prolonged sitting can moderate the positive effects of acute physical exercise breaks.

The present study offers several strengths and novelties that contribute to our understanding of the acute effects of physical exercise breaks on cognitive performance, cortical hemodynamics, and microvascular status. First, the study employed a randomized crossover design with pre‐ and post‐comparison, enhancing the robustness of our findings by minimizing biases (e.g., differences related to the individual)^[^ ^7c^ ^]^ and controlling for potential confounding variables. Second, our approach considered different levels of analysis by combining assessments of cognitive performance (i.e., RT, ER, and IES), cortical hemodynamics (i.e., cerebral activation and effective connectivity at both resting and task‐based states), and microvascular status using retinal vessel analysis (i.e., CRAE, CRVE, and AVR), and thus allows for a relatively thorough examination of the neurobiological processes of acute physical exercise breaks that may drive the positive effects on measures of cognitive performance. Third, our design includes three experimental conditions (i.e., vigorous‐intensity cycling, moderate‐intensity cycling, and prolonged sitting control) and three Stroop task conditions (baseline Stroop, color‐dual task, and lexical‐dual task). Such design allows us to identify how different exercise intensities and domains of the cognitive task influence the effects of acute exercise breaks on behavioral, retinal microvascular, and neurobiological outcomes. Fourth, we used LMMs^[^ ^68^ ^]^ for data analysis, which offer advantages over traditional methods by accounting for both fixed and random effects. This approach enhances precision in repeated measures, ensuring robust findings that reflect the simulated real‐world impact of acute physical exercise breaks. Fifth, we investigate the potential neurobiological mechanisms of the positive associations between microvascular parameters, brain functions, and cognitive performance by mediation analysis which advances our understanding of candidate mechanisms that can explain the positive effects of acute physical exercise breaks on cognitive performance during periods of prolonged sitting on which the direct empirical evidence is currently scant.^[^ ^11^, ^29^ ^]^

A further strength of our study is the relevance of our study findings to inform public health initiatives aimed at promoting physical exercise and reducing time spent on sedentary behaviors (especially mentally passive screen‐based sedentary activities such as non‐educational TV viewing) among college students. By demonstrating the benefits of acute physical exercise breaks on different indicators of cognitive and brain health, our study may help inform educational institutions seeking to optimize learning environments. However, implementing these acute physical exercise break interventions in practical settings is likely to face some challenges which include, the programming of the acute physical exercise breaks (e.g., exercise type and intensity) and the feasibility (e.g., academic scheduling conflicts and concerns that acute physical exercise breaks may disrupt classroom routines – but see for an example – https://drtejaspatel.com/in‐japan‐must‐exercise‐at‐work/). Addressing these requires a multidisciplinary approach involving educators, health professionals, and policymakers to integrate physical activity relatively seamlessly into students' daily lives.

## 5 Conclusions

Our study sheds light on the acute effects of physical exercise breaks on cognitive performance in college students, as well as some neurobiological mechanisms that potentially contribute to the acute physical exercise‐induced cognitive performance benefits – namely cortical hemodynamics and microvascular status. Our findings suggest that incorporating acute physical exercise breaks during periods of prolonged sitting (e.g., in classroom settings) can lead to immediate improvements in executive function, accompanied by changes in retinal vessel diameters, cortical activation and efficient connectivity, all of which indicated improved microvascular and brain function. These findings underscore the importance of integrating acute physical exercise breaks into daily routines, especially in environments with a high level of sedentary behavior like educational institutions, office work (e.g., reception, call centers, computer programming, legal sectors), and transportation (e.g., driving, flying). Future research should aim to elucidate potential dose‐response relationships between acute physical exercise breaks and cognitive performance, including but not limited to the optimal duration and type (e.g., cycling, walking, running), to thoroughly assess the versatility and efficacy of acute physical exercise interventions in bolstering cognitive performance and brain health. Future research should also investigate the long‐term effects of regular physical exercise breaks on cognitive function, and indicators of brain health, as well as investigate potential neurobiological mechanisms underlying the potential physical exercise‐induced improvements of cognitive performance.

## Conflict of Interest

The authors declare no conflict of interest.

## Author Contributions

Q.Y. and Z.H.Z. contributed equally to this work as joint first authors. L.Y.Z., S.L., K.I.E., C.H.H., A.F.K., F.H., Q.Y., and Z.H.Z. conceptualized and designed the research. Q.Y. and Z.H.Z. performed the formal analysis and data curation. Q.Y. and Z.H.Z. wrote the original draft. S.L., K.I.E., B.C., D.M.P., C.H.H., A.F.K., R.S.F., T.L.A., S.P.M., K.K., T.I., D.A.R., M.H., D.M., A.O.W., F.H., and L.Y.Z. reviewed and edited the manuscript. M.J.H. and J.K. conducted the investigation. L.Y.Z. supervised the project and managed project administration.

[Correction added on November 27, 2024 after first online publication: “Author Contributions” has been updated in this version.]

## Data Availability

The data that support the findings of this study are available on request from the corresponding author. The data are not publicly available due to privacy or ethical restrictions.
